# Better Late Than Never: Definitive Anatomic Repair of Dextro-Transposition of the Great Arteries

**DOI:** 10.1016/j.jaccas.2024.102269

**Published:** 2024-02-27

**Authors:** Asma Bahrami, Stefan P. Kostelyna, Samuel J. Dugger, Christopher R. Broda, Peter R. Ermis, Christopher A. Caldarone, Wilson W. Lam

**Affiliations:** a Department of Medicine, Baylor College of Medicine, Houston, Texas, USA; b Department of Pediatrics, Baylor College of Medicine, Houston, Texas, USA; c Division of Pediatric Cardiology, Department of Pediatrics, Baylor College of Medicine, Houston, Texas, USA; d Division of Cardiology, Department of Medicine, Baylor College of Medicine, Houston, Texas, USA; e Division of Cardiothoracic Surgery, Department of Surgery, Baylor College of Medicine, Houston, Texas, USA

**Keywords:** adult congenital heart disease, heart failure, heart septal defects, thoracic surgery, transposition of the great arteries, ventricular

## Abstract

We present a case of anatomic repair of dextro-transposition of the great arteries (d-TGA) with ventricular septal defect (VSD) in a 55-year-old man who presented with acute heart failure. This case highlights the importance of multimodal imaging and multidisciplinary involvement in developing a comprehensive surgical and medical plan for adults with congenital heart disease. We think this is the oldest reported patient undergoing anatomic surgical repair of d-TGA with VSD.

## Graphical abstract

**Figure:**
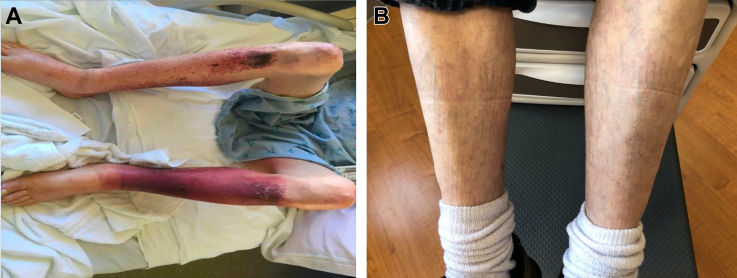

## History of Presentation

A 55-year-old man with dextro-transposition of the great arteries (d-TGA), ventricular septal defect (VSD), pulmonary stenosis (PS), and severe aortic valve regurgitation was admitted for decompensated heart failure. His congenital heart defect remained unrepaired after a palliative atrial septectomy in infancy. He followed up with a general cardiologist and was told he was not a candidate for further surgical intervention. He developed several sequelae of his unrepaired defect, including biventricular systolic and diastolic dysfunction, atrial fibrillation, chronic kidney disease, and polycythemia secondary to chronic hypoxemia. His baseline oxygen saturations ranged from 70% to 80% on room air. His disease was classified as NYHA functional class III. He was on furosemide, spironolactone, and metoprolol succinate. He was not on angiotensin-converting enzyme (ACE) inhibitor, because its use was limited by hypotension.

**Learning Objectives**

• To review the invasive and noninvasive workup of d-TGA with VSD.
• To understand what definitive surgical operations exist to treat d-TGA.
• To understand the importance of ACHD-specific care in adults with congenital heart disease.

**Figure 1:**
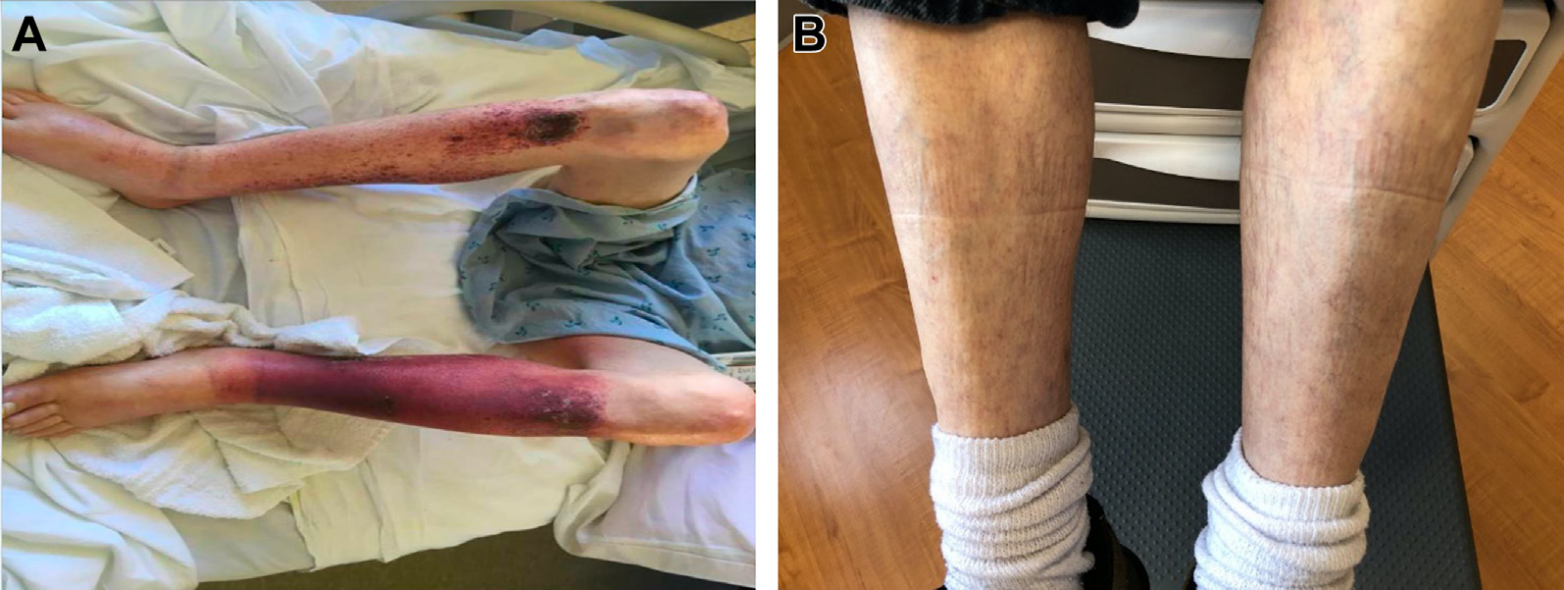
Cardiac Cachexia and Stasis Dermatitis With Subsequent Resolution Bilateral lower extremities of patient while in acute-on-chronic heart failure, NYHA functional class III, with cardiac cachexia and stasis dermatitis (A), with subsequent resolution after surgery (B).

His presenting vital signs were as follows: heart rate 104 beats/min, blood pressure 96/54 mm Hg, and oxygen saturation 76% on room air. His admission physical examination was notable for jugular venous distension, IV/VI systolic ejection murmur at the left upper sternal border with radiation to the lung fields, III/VI decrescendo diastolic murmur at the left lower sternal border, bounding pulses, and a sternotomy scar. Other pertinent findings included diminished breath sounds in the bilateral bases, hepatomegaly, cyanosis, clubbing, and lower extremity edema with stasis changes (Figure 1). He was admitted to the hospital in acute decompensated heart failure.

## Past Medical/Surgical History

Past medical history included d-TGA and VSD diagnosed after birth. He underwent Blalock-Hanlon atrial septectomy at the age of 18 days.

## Investigations

**Figure 2:**
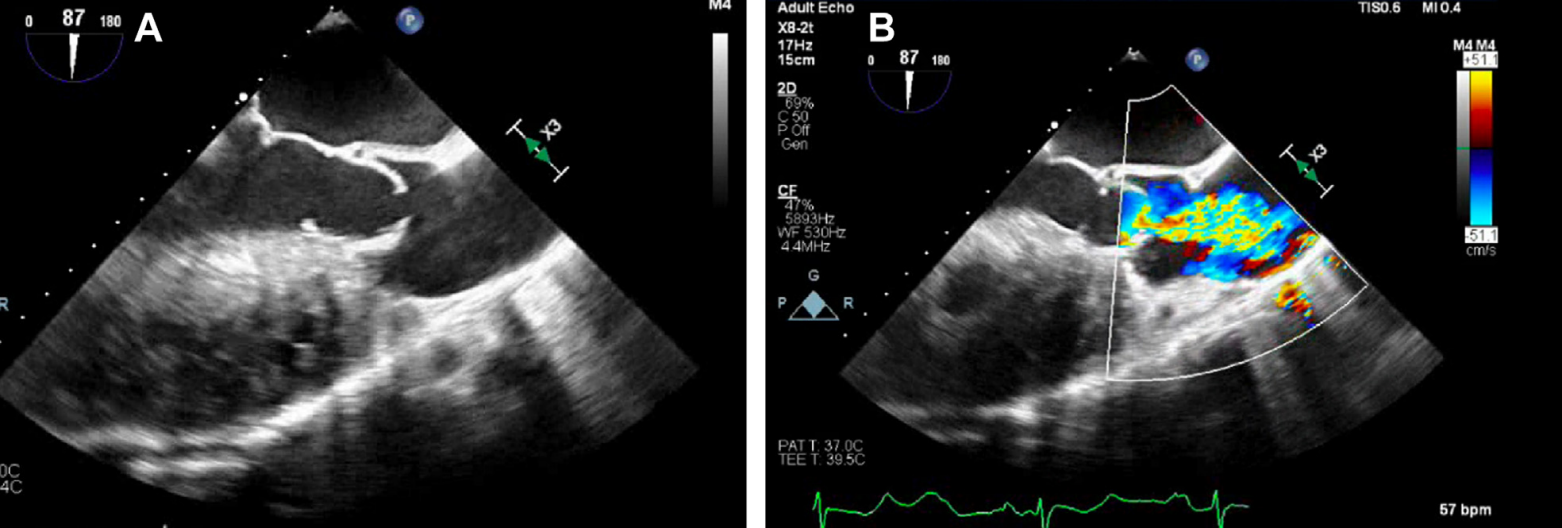
Transesophageal Echocardiography With Severe Pulmonary Stenosis This figure includes the transesophageal echocardiographic image depicting pulmonary valve doming with severe pulmonary stenosis in the patient before surgery. (A) 2-dimensional image; (B) color Doppler.

**Figure 3:**
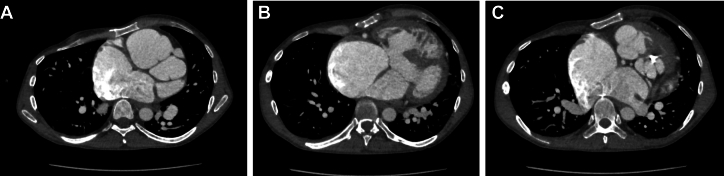
Computed Tomographic Angiography Depicting Variant Coronary Anatomy Computed tomographic angiography delineated the variant coronary artery anatomy. The left circumflex artery originates from the right coronary artery with focal aneurysm of the proximal left anterior descending artery at the first diagonal. (A) The left coronary aneurysm. (B) The large inlet ventricular septal defect. (C) The atrial septal defect and right coronary and left circumflex arteries.

The patient’s electrocardiogram showed first-degree atrioventricular block (PR interval 230 ms), interventricular conduction delay (QRS duration 114 ms), and prolonged QTc (503 ms). Echocardiography revealed a subpulmonic left ventricular ejection fraction (LVEF) of 50% and a systemic right ventricular ejection fraction (RVEF) of 40%, significant subvalvar and valvar pulmonary stenosis (inadequate spectral Doppler to fully quantify), severe aortic regurgitation, severe aortic root dilation (6.0 cm in maximum dimension), and a large unrestrictive inlet VSD (Figure 2). Cardiac magnetic resonance imaging (MRI) showed interatrial communication, an inlet VSD, an enlarged left ventricle with LVEF 46%, flattening of the interventricular septum, significant right ventricular enlargement, and concentric hypertrophy with RVEF 31%, PS, aortic root dilation (6.0 × 5.9 cm), and significant aortic regurgitation (regurgitant fraction 64%). Computed tomographic angiography (CTA) showed aneurysmal dilation of the left anterior descending artery at the branching of the first diagonal with mild ectasia of the right coronary artery (Figure 3, Video 1). Once euvolemic, he underwent right and left heart catheterization, which revealed normal pulmonary vascular resistance (PVR) and mildly elevated filling pressures (Table 1).

**Table 1:** Pressure Data From Right- and Left-Side Catheterization

	Systolic, mm Hg	Diastolic, mm Hg	Mean, mm Hg	Saturation, %
Superior vena cava/right atrium			17	35
Right ventricle	106	8	20	
Pulmonary artery	40	25	30	75
Left atrium			16	
Left ventricle	103	11	20	
Aorta	105	43	66	65

Cardiac catheterization showed Qp:Qs 2:1. Pulmonary vascular resistance was 2.1 WU.

## Management (Medical/Interventions)

Presenting in decompensated heart failure made anatomic surgical repair prohibitively high-risk. He was admitted to the cardiac intensive care unit for intravenous diuresis and inotropic support to optimize volume status. His preoperative hospital course was complicated by atrial fibrillation with rapid ventricular rate and nonsustained ventricular tachycardia, controlled with amiodarone. At our adult congenital heart disease (ACHD) multidisciplinary case conference, we decided to proceed with anatomic repair because his normal PVR made biventricular repair feasible.

**Figure 4:**
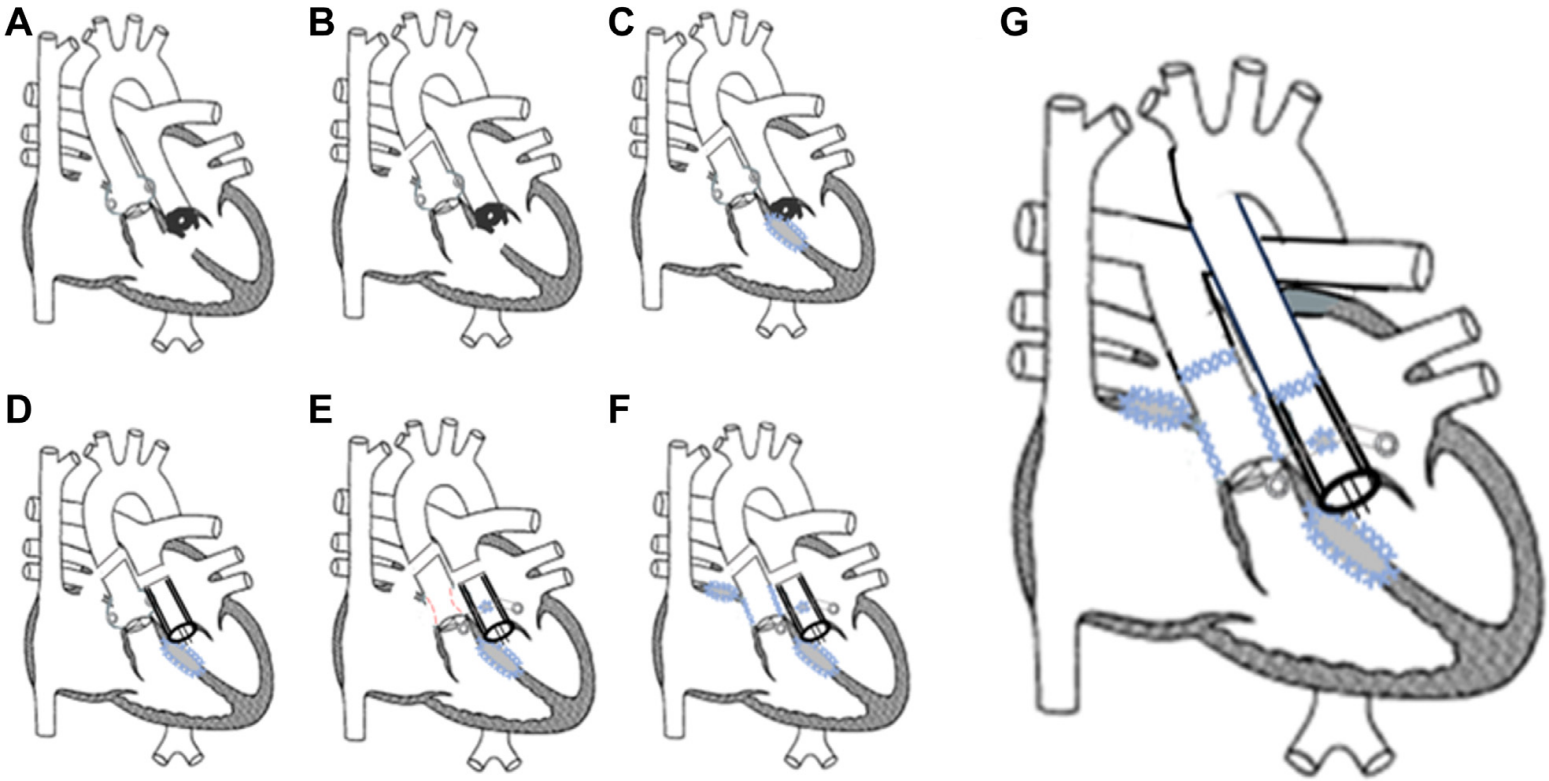
Modified Mullins Diagram Depicting Surgical Steps Stepwise depiction of the patient’s operation. (A) preoperative anatomy; (B) transection of aorta and visualization of coronary ostia; (C) transatrial closure of ventricular septal defect with Bard Sauvage implant; (D) neo-aortic root replacement and aortic valve replacement (Bentall) with 23-mm St Jude composite bileaflet valve; (E) mobilization of coronary buttons and implantation into neo-aortic root using Cabrol modification with 10-mm Gelsoft graft; (F) closure of coronary button harvest site and atrial septal defect closure with 5 × 8 cm Cardiocel patch; (G) arterial switch with mobilization of main pulmonary artery posteriorly and to the right of aorta (postoperative anatomy).

Once euvolemic, he underwent a complex operation that included Bentall replacement of the pulmonary (neo-aortic) root a with a 23-mm St Jude bileaflet valve, translocation of the coronary arteries to the neo-aorta with the use of a modified Cabrol graft, patch repairs of the atrial septal defect and VSD, and arterial switch (without Lecompte maneuver). After harvesting the coronary buttons, repair of the right ventricular outflow tract and neo–pulmonary valve was achieved with the use of the patient’s native valve tissue. Use of a Cabrol graft for coronary translocation was required owing to severe dilation of the aortic root at the sinus of Valsalva and dilated proximal coronary arteries, and the modification was chosen over the traditional Cabrol technique to avoid tension. Another consideration was the older age of the patient, making mobilizing the coronary arteries and reanastomosing them to a smaller root technically difficult. Diagrammatic depictions of the steps of the patient’s surgery are shown in Figure 4.

The procedure had no complications, and the patient weaned off cardiopulmonary bypass without difficulty. Intraoperative transesophageal echocardiography demonstrated competent and unobstructed pulmonary and aortic valves with a 1–2-mm residual VSD. Total aortic clamping time was 256 minutes, and total bypass time was 367 minutes. He was extubated on postoperative day 1, transferred to floor status on postoperative day 10, and discharged home on postoperative day 12. Preoperative LVEF was 46% and RVEF 31%. Postoperative LVEF was 46% and RVEF only mildly depressed after the surgery.

## Discussion

We present a rare case of d-TGA, VSD, and PS presenting in an older male as decompensated heart failure. Diagnostic work-up revealed aortic root dilation complicated by severe aortic regurgitation treated with definitive repair. This case highlights the importance of multidisciplinary care in managing complex adult congenital heart conditions.

In patients with unrepaired isolated d-TGA, the mortality rate is 89% at 12 months.^1^ In comparison, in patients with a concurrent VSD the average life expectancy is 22 months.^1^ It is rare for a patient to survive over 50 years. In this case it was likely possible because of his large VSD, surgically created atrial connection, and his pulmonary stenosis, which limited pulmonary overcirculation (Qp:Qs 2:1) and created favorable pulmonary artery pressures. However, this case highlights the importance of ACHD specialists evaluating these patients before development of the sequelae seen in this case.

Surgical correction for patients with d-TGA, VSD, and PS most commonly consists of the Rastelli procedure, involving baffling of the VSD from the left ventricle to the aorta and creation of a conduit from the right ventricle (RV) to pulmonary artery (PA). Ideal surgical candidates have a straight pathway from the left ventricular apex to the aortic valve to minimize right ventricular compromise.^1^ Other surgical repair techniques for d-TGA include the arterial switch operation, atrial switch operations (Mustard and Senning), the Nikaidoh procedure,^2^ and the réparation à l’etage ventriculaire procedure. The atrial switch operations are no longer the preferred technique for treatment owing to long-term complications.^2^

Imaging in d-TGA is necessary to establish the diagnosis and define anatomy and functionality, including hemodynamic parameters and the degree of mixing of pulmonary and systemic circulations. In children, echocardiography is sufficient in most cases, for both preoperative and postoperative assessment.^2^ In adults, although echocardiography is often the first imaging modality pursued preoperatively, cardiac MRI is preferred for evaluating the RV because echocardiography is often limited due to poor acoustic windows. Echocardiography also correlates poorly with cardiac MRI on key measurements, such as right ventricular volume.^3^ MRI can be helpful in postoperative evaluation to assess conduit and baffle patency and the morphology of the coronary arteries, aorta, and PA. In our patient, MRI allowed visualization of the feasibility of both an RV to PA connection and a left ventricle to aorta connection. MRI can also be useful for estimating shunt fraction in overcirculation to predict if PVR is prohibitive. Cardiac CTA, which can provide information about anatomy and ventricular function but is unable to assess flow, is generally reserved for patients with contraindications for MRI. CTA is sometimes useful for evaluating coronary anatomy when not well visualized with other modalities.

Adult patients with complex congenital heart defects require close follow-up. Long-term complications of palliative and corrective surgeries are frequent, requiring rhythm monitoring and surveillance imaging. For example, common complications of the Rastelli procedure include dysfunction of the RV-PA conduit, potential for residual VSD, and baffle obstruction.^3^ In addition, these patients are at higher risk of biventricular dysfunction, arrhythmias, and sudden death. Access to an ACHD center is important for these patients for both treatment decisions and follow-up and is associated with a reduction in mortality that is more significant for patients with more complex disease.^4^ Our patient was seen at non-ACHD centers and told on multiple occasions that surgical repair was not feasible. His distance from an ACHD center served as one barrier to receiving a more complete evaluation earlier in life. Those living a greater distance from an ACHD center face longer travel, making it more difficult to schedule appointments, and tend to have more sociodemographic disparities.^5^ These factors led to a delay in his repair until age 55 years, whereas most Rastelli procedures are performed between the ages of 6 months and 40 years, with 60% being completed before the age of 5 years.^6^ We think our case documents the oldest anatomic repair for d-TGA/VSD.

## Follow-Up

He was seen in the ACHD clinic 1 month, and most recently 3 years, after surgery with a favorable postoperative course. He weaned off all medications (except warfarin for his mechanical valve), with restoration of normal heart size and function. Also, despite the lack of Maze procedure, permanent pacing, or antiarrhythmic medication, he remains in sinus rhythm. This demonstrates that volume unloading, improvement in cardiac pressure, and restoration of normal saturations can be sufficient to address atrial arrhythmias in the correct clinical context. He remains physically active and engaged in house projects without cardiac symptoms, consistent with NYHA functional class I.

## Conclusions

Complex congenital heart defects require long-term surveillance at a specialized ACHD center. Multimodality imaging and multidisciplinary input is key to developing treatment plans. We think this is the oldest anatomic repair for d-TGA with VSD, at 55 years, facilitated by our multidisciplinary approach and made possible by protected PVR.

## Funding Support and Author Disclosures

The authors have reported that they have no relationships relevant to the contents of this paper to disclose.

## Appendix

Supplemental Video 1 Computed tomographic angiography of aneurysmal coronary arteries
